# The role of cognitive dysfunction in the symptoms and remission from depression

**DOI:** 10.1186/s12991-015-0068-9

**Published:** 2015-09-22

**Authors:** Xenia Gonda, Maurizio Pompili, Gianluca Serafini, Andre F. Carvalho, Zoltan Rihmer, Peter Dome

**Affiliations:** Department of Clinical and Theoretical Mental Health, Faculty of Medicine, Semmelweis University, Kutvolgyi ut 4., Budapest, 1125 Hungary; Laboratory for Suicide Research and Prevention, National Institute of Psychiatry and Addictions, Budapest, Hungary; MTA-SE Neuropsychopharmacology and Neurochemistry Research Group of Semmelweis Univeristy and the Hungarian Academy of Sciences, Budapest, Hungary; Department of Neurosciences, Mental Health and Sensory Organs Sant’Andrea Hospital Sapienza University of Roma, Rome, Italy; Department of Neuroscience, Rehabilitation, Ophthalmology, Genetics, Maternal and Child Health (DINOGMI), Section of Psychiatry, University of Genoa, IRCCS San Martino, Largo Rosanna Benzi 10, 16132 Genoa, Italy; Translational Psychiatry Research Group, Department of Clinical Medicine, Faculty of Medicine, Federal University of Ceara, Fortaleza, CE Brazil

**Keywords:** Depression, Cognitive dysfunction, Hot and cold cognitions, Functioning

## Abstract

The disability and burden associated with major depression comes only in part from its affective symptoms; cognitive dysfunctions associated with depression also play a crucial role. Furthermore, these cognitive impairments during depression are manifold and multilevel affecting elementary and more complex cognitive processes equally. Several models from different directions tried to evaluate, conceptualize and understand the depth and magnitude of cognitive dysfunctions in depression and their bidirectional interactions with other types of depressive symptomatology including mood symptoms. In the current review, we briefly overview different types of cognitive symptoms and deficits related to major depression including hot and cold as well as trait- and state-like cognitive alterations and we also describe current knowledge related to the impact of cognitive impairments on the course and outcomes of depression including remission, residual symptoms, function, and response to treatment. We also emphasize shortcomings of currently available treatments for depression in sufficiently improving cognitive dysfunctions and point out the need for newer pharmacological approaches especially in cooperation with psychotherapeutic interventions.

## Background

Major depression is one of the leading causes of years lived with disability in the developed world, therefore exploring those factors contributing to sustained disability during depressive disorders is crucial. Studies show that in addition to mood symptoms, cognitive deficits associated with depression also play a major role in this [1].

## Review

### Cognitive symptoms during depression

ICD and DSM mention cognitive disturbances during depression only in general, most studies, however, describe a wide range of cognitive dysfunctions during the acute phase of depression. Research indicates that verbal and visual short and long-term memory, executive functions, psychomotor skills and attention are all impaired in depressed patients [2–7]. Results, however, are somewhat contradictory, in part because studies investigate patients of different age with different clinical characteristics, illness course and medication schemes with differing methodology. Furthermore, several studies observed sustained cognitive deficits also in patients in remission [4, 8]. Patients in everyday clinical practice also report complaints reflecting cognitive dysfunctions, and clinicians do not necessarily associate part of these complaints with cognitive dysfunctions; therefore, their recognition may be problematic (Table 1).

**Table 1:** Symptoms reflecting cognitive dysfunctions reported by patients in everyday clinical practice

Cognitive function/domain	Complaint	Impact on everyday life	Report of patient	Exploratory questions
Attention	Loses track of thoughts Cannot pay attention Difficulty concentrating	Cannot absorb information during conversation, watching a movie, or reading Cannot concentrate on tasks Lack of motivation	“I can’t concentrate” “It seems as if I don’t pay attention to anything”	Is it difficult for you to read a magazine or to work with data at your workplace, or pay attention to tv or in a conversation?
Memory	No short term memory Forgetfulness Cannot count	Does not remember everyday tasks Needs lists and notes Feels old Loses or forgets things At the workplace feels embarrassed and stupid	“I forget everything” “I forget tasks, dates, meetings, and it is especially inconvenient at my workplace”	Does it happen that you can not find your keys, you do not remember names, or what you need to buy, or you lose track of tasks to do at home or work?
Executive functions	Procrastination and delaying No self-confidence Inability to decide Cannot deal with more than one thing simultaneously	Not enough self-confidence for decisions Is afraid and anxious because of consequences Avoids making decisions, is anxious because of decisions and delegates them to others Procrastination	“I can’t decide in anything” “I don’t dare to decide” “I can’t make up my mind”	Is it difficult for you to make decisions at home or at work? Does it require a big effort to start or complete tasks at home or work due to this? How does this influence your everyday life?
Psychomotor speed	Brain is foggy Slowed movement Tiredness, lethargy	No energy The smallest task takes a long time Feels slowed down, and feels brain is not working Cannot think	“My brain feels blocked”	Do you feel your thinking is significantly slowed down?

Investigation of cognitive symptoms of depression was for a long time only secondary after mood symptoms, although cognitive impairments and abnormalities, spanning from symptoms affecting elementary neurocognitive functions through concentration problems, memory problems and inability to decide to negative automatic thoughts, dysfunctional attitudes and maladaptive schemata constitute an important part of depressive symptomatology [3], which also well reflects that multilevel cognitive disturbances intertwine the clinical picture of depression, and cognitive distortions typical of depressive thinking well describe the basic disturbance of cognitive processing (Table 2). Thus, although depression is considered a primarily affective disorder, we are increasingly aware that it is also paralleled by a very marked and clinically significant disturbance in cognitive functions [2]. At the same time pharmacotherapy of depression is aimed almost exclusively at alleviating mood symptoms, and pays only little attention to cognitive disturbance in depression in spite of the fact that cognitive symptoms are significant predictors not only of therapeutic response, but also of later everyday and psychosocial function, and they also play a prominent role among residual symptoms.

**Table 2:** Disorders of information processing in depression: frequent cognitive distortions [36]

Cognitive distortions and logical fallacies	
Dichotomous/all or nothing thinking	Absolute and black-and white thinking, refusing anything which includes any minor imperfections
Overgeneralisation	Generalization based on a single experience of failure, perceiving a single negative event as an endless series of failures
Negative filter/selective abstraction	Designating the whole situation as negative based on a single negative detail, disproportionate attention to negative details and ignorance for positives
Discounting positives	Successes, accomplishments and positive characteristics do not count
Jumping to conclusions	Negative conclusions in the absence of evidence; supposing without any basis that others will react in a negative way, the person continuously expects things to end badly
Magnification/minimization	Arbitrary and disproportionate maximization of own faults and negative events, arbitrary and disproportionate minimization of good characteristics or events
Emotional reasoning/logic	Reasoning based on emotions, treating negative emotions as facts, and drawing conclusions based on them
“Should” statements	Formulating expectations as primary motives, criticizing self and others with should and must not statements
Labeling	Identifying self with own mistakes, applying these labels instead of admitting and acknowledging own mistakes
Personalisation and blame	Holding self responsible for something the person has no control over or something the person is not responsible for, or blaming others ignoring own role

#### Hot and cold cognitive symptoms

Cognitive symptoms associated with depression primarily denote basically distinct and diverse phenomena; elementary neurocognitive changes during depression on the one hand, and pervasive depressive symptoms developing on the background of maladaptive schemata, dysfunctional attitudes and automatic negative thoughts on the other. Cognitive functions and dysfunctions cannot be separated from mood and emotional symptoms. In order to understand the versatile and multicolored cognitive alterations in depression we differentiate hot, or affect-laden and cold, or affect-independent cognitive functions. While disturbances of cold cognitions can primarily be identified with neurocognitive testing, disturbances of hot cognitions are often detected during conversation, although the two types of cognitive processes interact. “Cold” cognition indicates information processing independent of emotional influences, which can be detected with tests where the stimulus is emotionally neutral and the outcome of the test is not important from a motivational aspect [4]. Abnormalities of “hot” cognitions can be observed in tasks related to stimuli carrying an emotional valence. In depressed patients distortions of processing congruent with mood are reported in several cognitive domains, while in other domains abnormalities related to cognitive processing of reward and punishment have been described. Depressed patients give more negatively biased answers in tests related to emotional processing which also concern perception, memory, attention and working memory. Furthermore, depressed patients show altered performance in reward- and punishment processing which indicates an increased sensitivity towards negative feedback and a decreased sensitivity towards positive feedback, and decreased learning related to rewarding cues [4, 5, 9, 10]. Other authors, however, suggest that “cold” cognitive deficits observed in depression can also be explained in part by alterations in “hot” cognitive processing, that is, emotion-independent cognitive tasks in many instances become emotion-laden in depressed patients especially in case of feedback-based tasks. This is also reflected by the catastrophic reaction of depressive patients to perceived failure which means that following one mistake they show a higher error rate in the next trial [4, 5]. These well illustrate that elementary neurocognitive alterations play a central role in the manifestation of other depressive symptoms as well.

#### Cognitive neuropsychological theory of depression

One approach to depression is expressed in Beck’s cognitive model which views depression as developing on the bases of stable, self-reinforcing, maladaptive negative schemata and dysfunctional attitudes and attributional styles. Negative expectations contribute to the emergence of typical depressive thinking processes such as negative automatic thoughts, negative emotional biases or rumination, that is, contribute to abnormal “hot” cognitive processing in a top-down manner. During cognitive therapy for depression patients are in essence taught, by tasks involving working memory, inhibitory processes and problem solving, to be able to exert “cold” cognitive control over their top–down negative biases [4].

The modern cognitive neuropsychological model of depression is a reformulation and expansion of Beck’s cognitive model of depression with results from pharmacological studies and concerning elementary neurocognitive functions. This integrated approach postulates that during depression, beyond the above, due to a dysfunction in the monoaminergic neurotransmitter systems there is an alteration in the bottom–up processing of emotional stimuli resulting in negative perceptions, and thus negative biases and negative schemata result from the decreased monoaminergic modulation in neural circles in the background of emotional processing [4, 11] which is also supported by the observation that in healthy and depressed subjects bias concerning processing of reward-related and emotional stimuli can be influenced by manipulation of monoaminergic neurotransmission. The resulting dysfunctional negative schemata are themselves also capable of generating top–down biases manifested as negative expectations which sustain negative schemata [4]. By influencing bottom–up negative biases SSRIs decrease symptoms; however, this may only be successful if, with the help of these changes, patients make an effort to correct their dysfunctional cognitive processes by questioning and restructuring their top-down biases. This is also supported by the fact that pharmacotherapy and cognitive therapy are significantly more effective in combination compared to either method on its own [12].

#### Trait- and state-like cognitive deficits during depression

When reviewing cognitive deficits characteristic of depression it is important not only to distinguish between hot and cold cognitions, but also to differentiate between those cognitive functions occurring exclusively during depressed episodes and those observable also between episodes or even prior to the development of symptomatic illness. By identifying trait- and state-like cognitive alterations it would be possible to explore those cognitive characteristics and dysfunctions which are present even preceding the illness and can in many times be identified also in first-degree non-affected relatives and could therefore be considered as trait-like vulnerability markers. Furthermore, it is important to study and evaluate those residual cognitive symptoms which are present after the alleviation of affective symptoms during remission, since these profoundly and pervasively influence quality of life and function.

### The role of cognitive symptoms in illness course and function

In a prospective study in depressive patients it was established that in addition to lack of energy and problems of sleep, cognitive symptoms dominate during the course of depression, and cognitive symptoms are present during 85–94 % of the length of depressive episodes and 39–44 % of the length of periods of remission [13]. This indicates not only that this is a frequent complaint affecting a large portion of patients, but also that they negatively influence quality of life of depressed patients in the majority of time [13]. Deficits of executive function and verbal learning as well as certain other types of memory deficits can also be identified during euthymic periods in young adult patients [6, 14–16] and these are independent of clinical variables indicating severity of illness [3, 17]. Another study evaluated neurocognitive symptoms of depressed patients at baseline and after 6 months and at the time of follow-up 60 % of the investigated patients exhibited significant neurocognitive deficits [18]. Further studies indicated that with every episode there is a decrease in cognitive function and that interepisode cognitive function is related to the number of previous episodes [19, 20].

Cognitive impairment also plays a role in functional recovery from depression both in unipolar and bipolar disorder [18, 21]. Everyday function is often impaired even during remission, and in the background residual symptoms, comorbid conditions, false diagnoses and long-lasting cognitive impairment may play a role. Mood and affective symptoms on their own often fail to justify the impairment associated with depression [18], and the alleviation of affective symptoms is often not paralleled by an equal increase in daily function [22]. Impairments in several neurocognitive domains were shown to disrupt daily function. In a study in depressed patients it was found that 6 months after baseline neurocognitive function was strongly and significantly associated with functioning after controlling for residual depressive symptoms [18] which indicates that neurocognitive function plays a key role in functional recovery. Neurocognitive deficits may lead to the impairment of daily function in manifold ways, by deteriorating the chance of obtaining and sustaining a job, school and work related progress and promotion, academic and workplace productivity, sustaining a household and social and family relationships, as well as problem solving, and depression also impairs capacity for coping with the deleterious effects of the illness. Impaired coping and prolonged impairment affecting daily functions burdens family, friends and colleagues as well [2, 18, 23] thus deteriorating or diminishing social support over time, and therefore the patient is increasingly left to his own compromised problem solving and coping capacities which also further increases stress [18]. Therefore, neurocognitive deficits not only influence current function and quality of life, but can also predict long-term function. A study indicated that cognitive (mainly executive) functions evaluated at admission [24] predicted outcomes 4 months later in young depressives both related to severity of depression and work and social function. Improving cognitive functions also indicate functioning 6 months later [24] which suggests that improvement of neurocognitive function is associated with a greater likelihood of functional remission and thus emphasizes the importance of cognitive functions in the therapy of depression.

### Cognitive symptoms and pharmacotherapy of depression

Independently of the severity of symptoms neurocognitive deficits predict worse therapeutic response during SSRI pharmacotherapy in young and elderly patients also after correcting for severity of depressive symptoms [4, 24, 25]. However, interaction between cognitive impairment and pharmacotherapy is bidirectional; more severe cognitive deficits indicate not only worse response to SSRI pharmacotherapy, but treatment in many cases negatively impacts cognitive functioning. In one study one quarter of SSI-treated patients reported loss of creativity, and other cognitive side effects including concentration difficulties, loss of ambition, memory and problem solving capacity impairment [25]. Patients treated with newer antidepressants do better from a cognitive aspect compared to untreated patients, but still perform worse compared to healthy controls [26]. As we emphasized, cognitive disturbances are among the most common residual symptoms of depression in spite of treatment, which reflects that currently available antidepressants are not able to sufficiently improve cognitive symptoms of depression.

#### Serotonergic dysfunction and cognitive symptoms of depression

Acute tryptophan depletion leads not only to low mood in vulnerable subjects, but also cognitive dysfunction which, besides the well-known association between serotonergic function and major depression, also raises attention to the role of serotonergic dysfunction in the background of cognitive symptoms [27–29]. In line with this, pharmacotherapy aimed at the serotonergic system decreases depression-related deficits in cognitive domains and functions including episodic memory, working memory, attention and executive function [28, 30], although results are somewhat contradictory. Agents also including a noradrenergic component such as SNRIs or NRI improve certain cognitive functions during depression and SNRI therapy appears to be more effective with respect to cognitive functions compared to SSRI treatment [31, 32]. In spite of these, cognitive impairments associated with major depression in many cases also persist after affective symptoms disappear; therefore, sufficient treatment aimed at these symptoms is still lacking [28, 32, 33]. Results suggest that cognitive deficits resulting from serotonin depletion cannot be restored by antidepressants which act exclusively by blocking serotonin reuptake. A multimodal antidepressant, vortioxetine, besides inhibiting serotonin reuptake also exerts a direct pharmacological effect on various serotonergic receptors antagonizing 5HT_3_, 5HT_1D_ and 5HT_7_ receptors while exerting a partial agonist effect on 5HT_1B_ and an agonist effect on 5HT_1A_ receptors [34]. By directly impacting serotonergic receptor function in addition to blocking serotonin reuptake vortioxetine appears to restore memory deficits elicited by serotonin depletion according to preliminary results [28]. A preclinical rat study showed that unlike escitalopram and duloxetine, vortioxetine restored object recognition and spatial working memory deficits caused by serotonin depletion. Since occupancy of serotonin transporters is above 90 % in case of all three agents these results suggest that this effect is directly due to the effect of vortioxetine exerted on serotonergic receptors [28, 34]. In a double-blind clinical study in elderly depressive patients vortioxetine significantly improved performance in the Digit Symbol Test and the Ray Verbal Learning Test while duloxetine treatment only improved verbal learning parameters [35]. Performance on the Digit Symbol Test reflects the function of several cognitive domains and is a measure of processing speed, executive function and attention, therefore while effect of duloxetine on cognitive function was mainly mediated by verbal learning and memory, vortioxetine, which improves cognitive functions on both tests delivered a broad effect due to the complex impact on neurotransmitters playing a role in cognitive function [34]. Path analyses also indicated that two-thirds of the effect of vortioxetine on cognitive function is a direct effect and is not due to an improvement in the mood symptoms of depression.

## Conclusions

Depression profoundly and fundamentally changes perception of and interaction with the environment and pervasively impacts elementary and complex neurocognitive processes which play a role in these. Furthermore, the effect of depression on cognitive function determines daily function in the long term and also influences to which degree patients are capable of psychotherapy and psychotherapeutic improvement [4]. Persistent cognitive dysfunction is also important clinically, because it decreases coping capacities and influences therapeutic compliance and cooperation as well [6]. The results of studies show that during major depression cognitive deficits profoundly influence therapeutic response, risk of relapse, as well as function and quality of life, and in several cases they persist between two acute episodes during remission as well. Cognition is thus a key target in the treatment of depression especially with respect to early recognition and intervention, since by the improvement of cognitive symptoms not only functional decline, but also risk of relapse can be decreased [17]. Psychotherapeutic interventions aimed at improving cognitive dysfunctions, such as adaptive or compensatory strategies targeted at frontosubcortical functions, diaries, memory improvement, or formal cognitive remediation and neurorehabilitation programs not only decrease symptoms, but also disability associated with depression. Therefore, on the one hand, such psychosocial, occupational or cognitive programs are needed which target cognitive deficits associated with depression at multiple levels, while on the other hand we need to pay more attention to these symptoms also during pharmacotherapy.
